# Voice coil-based scanning probe microscopy

**DOI:** 10.1186/1556-276X-7-332

**Published:** 2012-06-21

**Authors:** Petr Klapetek, Miroslavmvaltr@cmi.cz, Václav Duchoň, Jaroslav Sobota

**Affiliations:** 1 Czech Metrology Institute, , 638 00, Okružní 31, Brno, Czech Republic; 2 Institute of Scientific Instruments, Academy of Sciences of the Czech Republic v.v.i., 612 64, Královopolská 147/62, Brno, Czech Republic

**Keywords:** SPM, voice coil, interferometry

## Abstract

We present a novel system for large-area scanning probe microscopy (SPM) measurements based on minimum counter-force linear guidance mechanisms, voice coils, interferometers and fuzzy logic-based feedback loop electronics. It is shown that voice coil-based actuation combined with interferometry can be a good alternative to piezoceramic positioning systems, providing fast and still sufficient, precise displacements which range from nanometers to millimeters. Using fuzzy logic feedback control, it can be actuated even with only a few low-cost components, like a cheap single-chip microcontroller. As the final positioning resolution can be made independent on the electronics output resolution, the system can reach high positioning resolution even on very large scan sizes. This is a key prerequisite for developing novel generations of SPMs that would combine, in a very large range, with high-speed imaging.

## Background

Scanning probe microscopy (SPM) techniques and, namely, atomic force microscopy (AFM) are key tools for dimensional measurements at the nano- and microscale. Due to their simplicity, well-defined operation and the possibility to split the motion into three independent axes, they also became a typical metrological traceability source for dimensional measurements on nanostructured surfaces, which is very important.

In SPM techniques, piezoelectric transducers are typically used in order to convert voltage into a displacement in a positioning system. Scanners are formed directly by piezoelectric components, or they are constructed as a combination of guidance systems (e. g. flexure based) and piezoelectric components. The *xy* range is usually in the order of tens of micrometers. More than 100 *μ*m is rarely obtained for a single positioning system. The *z* range spans a few micrometers only in most of the microscopes, as the structures that are analyzed by SPM are usually relatively flat. The needs for the resolution of the *z* axis positioning system are extreme, typically needing a resolution of less than a nanometer. The *xy* axis system can be of slightly lower resolution, ranging from sub-nanometer values in high-resolution systems up to some 10 to 20 nm in large-range or low-cost systems.

There are, however, some principal disadvantages of using piezoceramic components. First of all, they exhibit many unwanted mechanical and electrical properties (creep, hysteresis, aging, etc.). These effects can be partially compensated using feedback loop-based, independent displacement sensors or using charge drive instead of voltage drive [1]. Second, they have a limited actuation range, and they need a high voltage for their operation. This limits the use of piezoceramic components in many applications, namely in the field of coarse positioning. Today, most SPM systems need to have both a coarse positioning system (using e. g. servomotors) and a fine positioning system, using piezoceramic components.

As an alternative to piezoceramics, electromagnetic positioning systems known as voice coils can be used for the *xy* positioning, similarly as in other fields of technology [2-4]. In principle, this can have many advantages. The system is no longer limited to the small range of piezoelectric components. No coarse positioning may be needed if the system has a traveling range of at least a few millimeters. Voice coil systems are also a solution of how to build long-range scanning probe microscopes that would have a much larger measuring range but still preserve nanoscale accuracy. Such systems are already reported in the literature [5-9], are even available in the market [10], and often use voice coils for their actuation.

In this article, we present an algorithm for the actuation of a voice coil-based *xy* scanning system. Namely, we discuss the effects of the counter-force on the performance and dynamic range of voice coil systems based on flexure guidances. The algorithm was implemented on two different voice coil-based positioning systems: one already presented in the literature recently [5] and one completely new, trying to get optimum speed and performance of the presented approach. In this article, this new design, which features, namely, compact size and higher speed for data acquisition, is presented. Note that in this article, we focus on *xy* positioning only, while the complete SPM performance is a combination of the *xy* positioning stage and the *z* stage performance, mechanical construction of all the other components (sample holder, coarse approach mechanism) and probing system performance (cantilevers, light lever based or other force detection, etc.).

## Methods

### Experimental arrangement

**Figure 1:**
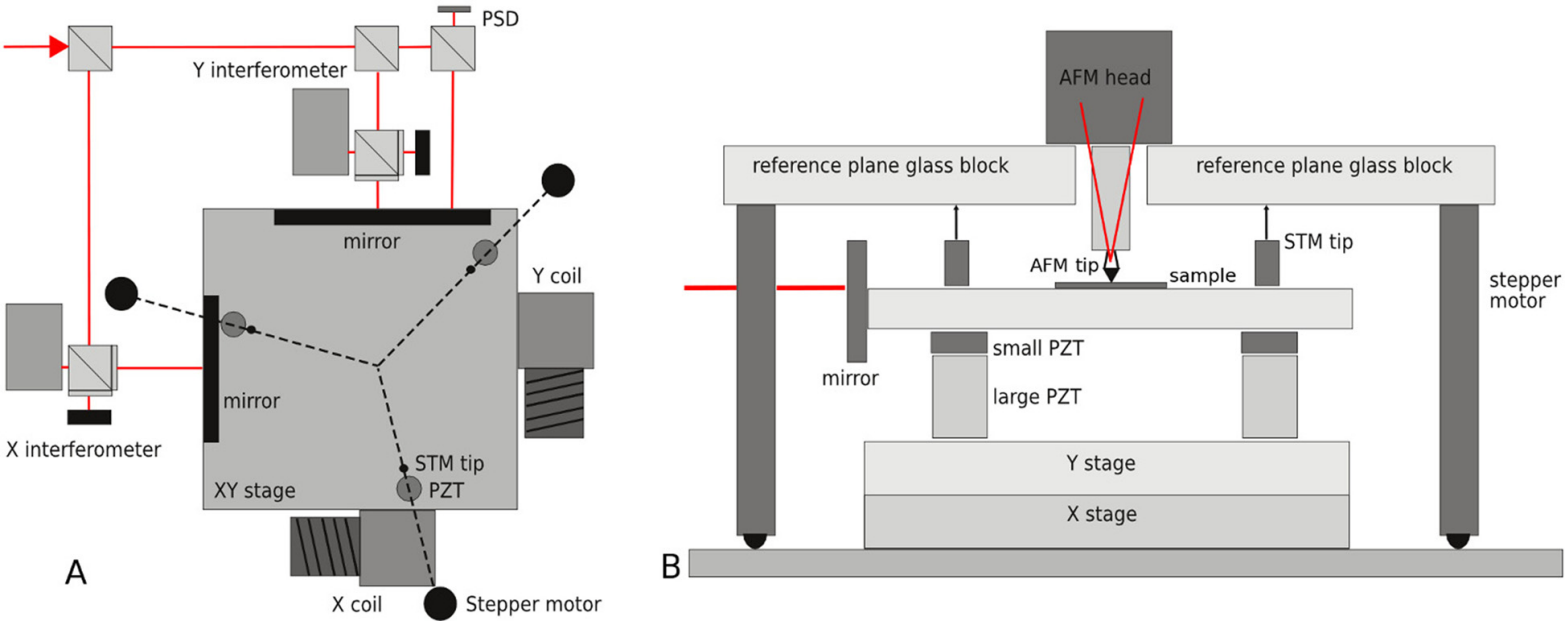
**Schematics of the crossed roller bearing-based stage.** (**A**) The *xy* motion system (position of the three reference plane sensors and actuators shown as well) and (**B**) the reference plane system schematics (2D plot; note that in practice, the system has a threefold symmetry as seen from the top view of A).

Our first system, already described in detail in [5], is a home-built, long-range system based on commercial crossed roller bearing stages combined with piezoceramic actuators used to compensate the imperfections of the bearing mechanism. Three interferometers are used for all three axis translation monitoring and feedback. For stage rotation monitoring (axis normal to sample surface), an autocollimator is used. For non-planarity compensation and two more axis rotation compensations (axes parallel to sample surface), an optical quality reference plane and a set of tunneling current sensors are used. These sensors are used to ensure that the planarity of the *xy* motion can be referred to a measurable object (reference plane) and not only to the crossed roller bearing stage (for more details, see [5]). The construction of the system is illustrated in Figure 1 and, in more detail, described in [5]. The thermal stability of the system is assured using an active stabilization box with digitally controlled, thermally stabilized water.

**Figure 2:**
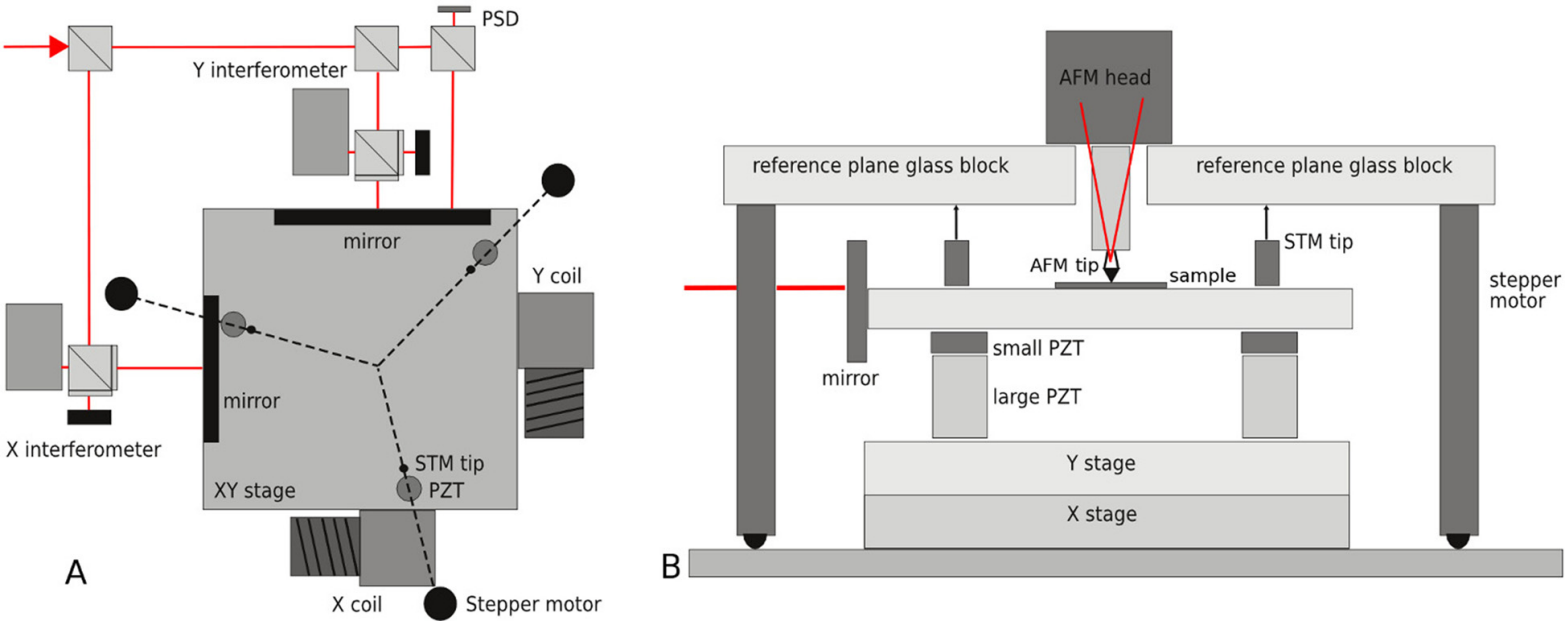
**Schematics of the flexure-based stage.** (**A**) Flexure-based stage mechanical construction. (**B**) Compact interferometer schematics.

**Figure 3:**
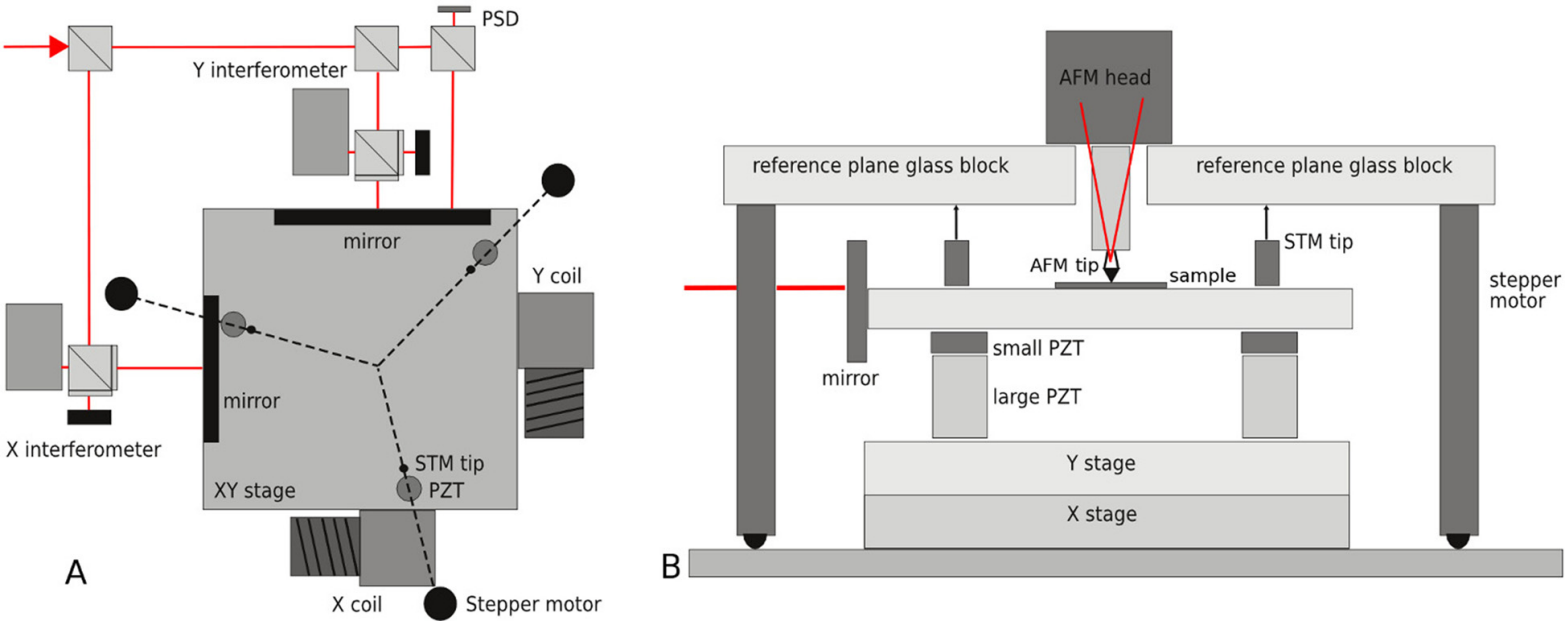
**Mechanical parts of the*xy*positioning system.** Two different mechanisms of linear guidances are used: (**A**) crossed roller bearing stages and (**B**) low-stiffness flexures. Note that only the guidance systems with voice coils are shown here; in the complete setup, these are enclosed and hidden by interferometers, sample holder and AFM head.

The second system was built to optimize the scanning speed and to make the system more compact. The main constraints were, therefore, size, guidance planarity and minimal counter-force. In order to get maximum speed, we tried to make the moving part as light as possible. For such a construction, we could not use bearing stages any more, so we had to concentrate on building a very light flexure-based stage. As we wanted to use force feedback (see below), it is important that the flexures are as weak as possible, getting the lowest possible resonance frequency of the system. Ideally, we would like to have a flexure system with no counter-force at all over a size of a few millimeters, but this was not realistic. The prototype construction, shown in Figure 2, was based on tiny phosphor bronze flexures combined with cuprextite elements glued and soldered together. As a motor for both axes, a 2D voice coil from a DVD head was employed, connected with the moving part of the stage by thin, but rigid wires. Photographs of both systems can be found in Figure 3.

For both *xy* positioning systems, the same atomic force microscope head was used in determining the probe-sample distance and performing the *z*-axis motion (fine motion based on piezoceramic actuators and coarse approach based on a stick-slip actuator).

For illustrative measurements, a sample obtained during microchip manufacturing process was used. This microelectronic device represents typical measurands requesting large-scale measurements in long-range SPMs. Contact mode measurements were performed using standard contact tips from Nanosensors (PPP-CONTR series, Neuchatel, Switzerland).

### Voice coil feedback control

We have used the same controller for both systems, based on simple custom-built electronic boards. As a source of feedback signal, we use the sine and cosine signals from a Michelson interferometer (which uses polarised light to obtain a quadrature signal). The whole feedback algorithm is implemented on a low-cost ATxmega controller (ATxmega128A1, Atmel Corporation, San Jose, CA, USA) that is able to perform a quadrature decoding via its specialized module, i. e. without the need of any additional computational power. As the interferometric signal detection would be the most demanding task otherwise, using the hardware support for the quadrature decoding, we can easily run the feedback loops for the two interferometers even on a relatively low-computational-power and low-cost device. The sine and cosine signals from the interferometers are used both in the analog form and in the digital form. The digital signal is obtained using a comparator circuit (comparing the analog signal to zero voltage, a standard operational amplifier TL074 is used) combined with a non-inverting amplifier to adjust voltage levels to a range of 0 to 3.3 V, which is the range of allowable voltage levels of the ATxmega device. The digital signal is used for the quadrature decoding, while the analog signal is used (after a similar adjustment of voltage levels) for a precise phase detection when the system is moving slowly. The decoding system is, therefore, able to perform a very fast detection of the number of fringes as well as a slow detection of the signal phase. As the microscope is moving relatively slowly while scanning (comparing to the circuit speed), this approach is sufficient for scanning speeds up to several millimeters per second (while recording both the number of fringes and phase) or several centimeters per second (while recording only the number of fringes). Note that only the fringe counting is used during the fast positioning, and the phase is used for the fine adjustment of the required position. The AFM measurement is performed point by point, so the system uses the phase to perform the fine adjustment at every measured position.

Even if the mechanical properties of both *xy* positioners are rather different (mass, maximum force, presence of a counter-force), we have developed a universal algorithm that is able, after proper parametrisation, to control both of them. The key task is how to determine the voltage that should be sent to voice coils if we know the actual position and speed from the interferometers. There are two extrema that we need to reach: 

· For the crossed roller bearing stage, there is almost no counter-force observed, except for small effects caused by irregularities of the bearings. If we leave the system with no force produced by the coils, it stays in place, or if in motion, it moves with almost constant velocity in time scales typical for our control loop.

· The flexure system has an observable counter-force, trying to move the system to the center of its range. Even if the force is significantly smaller than the maximum force that can be obtained from the coil (up to 50% of it), it highly affects the system response to an impulse.

Besides the counter-force effect, we need to address different masses and related high inertia of the crossed roller bearing stage. The inertia of the flexure system is relatively high even if it is compared to the force that can be produced by its coils. For such systems, however, the traditional proportional-integral-derivative control mechanism looks ineffective. An active deceleration is necessary for an optimum actuation, which means that we need to get a force of the opposite direction in order to stop at the desired location.

The developed algorithm has two independent parts. Their ratio during actuation can be controlled by a single parameter which is usually a constant, but it is different for each of the two systems. 

· The first part of the algorithm is based on a fuzzy logic approach. Here, two variables are used in controlling the voltage applied to the voice coils. The first variable is the position error (determined from the setpoint and the actual position value); the second one is the movement speed, including its direction (determined by the numerical differentiation of the position value). A lookup table is assembled to determine the output quantity (a number to be sent to the digital-to-analog converter (DAC)). A typical lookup table organisation is shown in Table 1. The *x* axis (horizontal with respect to the page orientation) is the position error, and the *y* axis (vertical) is the movement speed. The result to be sent to the DAC can be simply taken as an output value by a lookup process, or, to be more precise, the value can be interpolated from the neighbor values to get the output. The center of the table corresponds to the situation when there is no error in position and the speed is zero. Hence, no voltage is applied to the voice coil and no force is generated. The upper right and lower left corners correspond to a situation when the error is high and the direction is the proper one. Here, the applied force can even be zero, as the system moves by its inertia. In contrary, the upper left and lower right corners describe the opposite situation - large error but wrong speed direction; the system has to generate opposite force to correct this. In order to increase the feedback speed, the table is interpolated at the program start at much higher resolution and only a lookup process is performed. In this manner, the microprocessor needs only very few instructions to get the feedback signal.

· The second part of the algorithm is based on an integral term known from proportional-integral-derivative controller theory. This term compensates the counter-force of the guidance mechanism. Note that the integral term needs to work more slowly than the first part of the algorithm to prevent the mutual interference. This, in principle, limits the possibility of performing fast jumps over large distances. Fortunately, rapid and large changes of the position (e. g. going immediately from one corner of the image to the other) are not typical for SPM scanning and would be even senseless due to the limited speed of the probe-sample feedback loop.

The parametrisation of the whole algorithm can be performed via a few constants. The fuzzy logic part of the algorithm sensitivity is controlled by scaling constants for the position error and speed. The integral term in the second part of the algorithm has a time constant that can be adjusted. Finally, a mixing ratio between both parts is set up. As a result, we get a single number for each axis that is sent to a DAC (the built-in one on the ATxmega chip is sufficient) and then via a power operational amplifier TCA0372DP2 (ON Semiconductor, Phoenix, AZ, USA) fed to a voice coil.

**Table 1:** A typical lookup table organisation

	***x***				
***y***	–200	–160	–100	–61	0
	–160	–121	–40	0	61
	–100	–40	0	40	100
	–61	0	40	121	160
	0	61	100	160	200

The *x* axis (horizontal with respect to the page orientation) is the position error, and the *y* axis (vertical) is the speed of the motion. The appropriate cell value can be directly used to feed the DAC for a voice coil voltage.

## Results and discussion

There are three key questions that we would like to address in this section: 

· What is the positioning system precision, including systematic errors and feedback system resolution?

· What speed can the positioning system reach while being in feedback?

· Is the positioning system suitable for scanning probe microscopy imaging?

An actuator can, in principle, have many different errors. Here, we concentrate on two main problems: it can move in a different direction than expected and it can bend during the motion. The system guidance errors for the crossed roller bearing (CRB) stage and the flexure stage were evaluated using an interferometer and a digital autocollimator. The latter was based on a position-sensitive detector combined with a laser beam reflection from a mirror on the stage. For comparison, the same guidance errors were measured on a stack piezoceramic element representing a typical solution that is used in some microscopes (mechanical guidances are used only rarely in commercial AFMs) and also on a *xy* system of an older commercial AFM. The results are summarized in Table 2. The crosstalk (a parasitic motion in the second axis due to the motion in the first one) and the rotation (a dependence on the deviation of the orientation of the positioner’s moving part from the direction of the motion) were evaluated. We can see that the mechanical errors of both guidances are lower than those of piezoceramic solutions. A properly constructed piezoceramic AFM scanner will have probably better metrological parameters than the presented stack actuators; however, it can be seen that the presented approach is fairly within the limits of present microscope technology.

**Table 2:** Comparison of metrological properties of different systems

**System**	**Movement (nm)**	**Rotation (*μ*rad)**
CRB stage	0.4	0.005
Flexure stage	3.8	0.45
Commercial SPM	2	2
Stack piezo	27	40

Comparison of parasitic motion and rotation error for different actuators. Note that for the CRB stage, the parasitic motion could not be measured, as the system could not be disassembled; the values are taken from a single stage datasheet. For the flexure stage, it was measured with one axis fixed. For the commercial SPM, the estimate is taken from AFM measurements of a calibration standard.

The feedback algorithm, as run on the microchip, has itself a bandwidth higher than 100 kHz, which is far more than what we can get from the hardware parts used in the rest of the system. The final feedback speed is influenced, namely, by two other factors: the moving mass and the force that can be generated. The masses are 2 and 6 kg for the *x* and *y* axes of the CRB stage, respectively, while for the flexure stage, these are 16 and 20 g, respectively. The maximum force is 0.8N for the first stage and 0.02N for the second stage. As we can see, the smaller system could be approximately ten times faster, which was also observed in practice. While the large system needs some 300 ms to reach any position (with almost no dependence on the distance), the smaller one needs some 30 to 80 ms, depending on the distance (this is the effect of the proportional term of the algorithm).

As we have seen, the moving mass has a big influence on the feedback algorithm performance, and the algorithm is expected to be tuned with respect to it. In practice, this means that we need to keep the mass of the samples well below the mass of the moving part of the positioner, if we do not want to change the feedback parameters with every sample. Typically, we have scanned samples with a mass of up to 1/10 of the moving part of the positioner mass, which means some 200 g for the CRB stage and some 1.6 g for the flexure-based stage. Even if it might look as a small number, this is relatively easy, as the mass of our typical samples is very small - 1.6 g, which corresponds to a silicon wafer piece of some 10 cm^2^.

The big advantage of the feedback based on the force is that in the absence of the counter-force, the system is not dependent on its position. At every position, the system can reach the same accuracy, given only by the interferometric sensor accuracy. There is no need for large-bit depths of the DAC output to the coils as the force itself is not connected with the final resolution. This is a rather different system compared to the system using piezoceramic components, where the DAC bit depth is directly connected with the scanner resolution. Of course, when we have to produce a large compensation of the counter-force, we lose some of these advantages as the counter-force usually depends on the position. It is therefore important to preserve the counter-force as low as possible at the scanner design and manufacturing phase. The system resolution is based on the two interferometer and feedback loop imperfections; it is typically around 15nm for both systems, but it could be tuned even to some 5nm if the parameters were adjusted with greater care. Note that the resolution does not mean accuracy, which is determined by more factors, including laser stability, geometrical errors (like the Abbe error), interferometer non-linearities, etc. However, from the first estimates, it looks that the feedback loop noise determining the above resolution is by far the biggest uncertainty component of the whole positioning system uncertainty budget.

**Figure 4:**
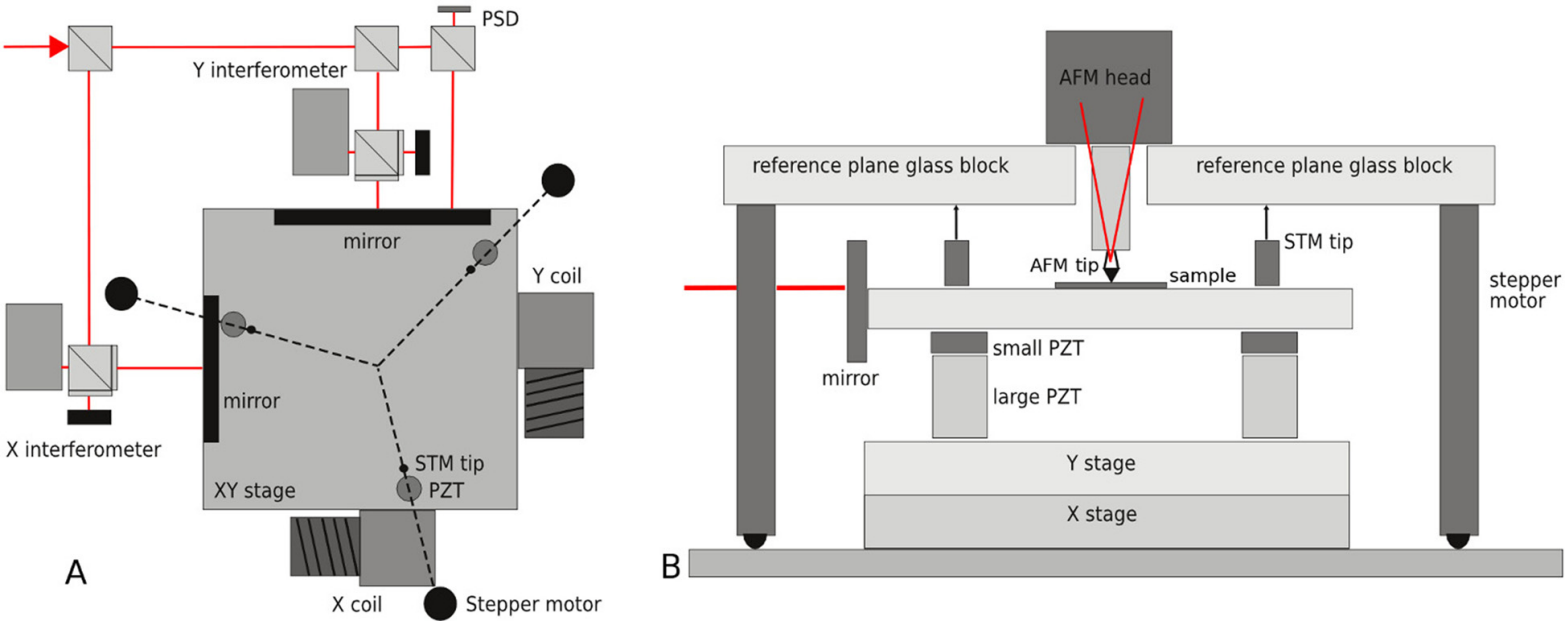
**Images of a microchip surface acquired using both systems.** (**A**) AFM measurement using CRB stage. (**B**) AFM measurement using flexure-based stage.

The suitability of the system for the SPM imaging is another important parameter. The fact that the positioning stage is able to move with a certain resolution and accuracy, it still does not necessarily implicate that it is suitable for these purposes. Some actuators used for the nano- and microscale positioning do not move smooth enough to enable a tip-sample feedback loop. In some cases, there are significant vibrations or acoustic noise during the motion. A typical example is a stick-slip actuator, which is often featuring both effects. As there is a rather independent and quickly randomly changing force applied on the stage by the voice coils in our system, it could be easily possible that our system has similar errors. The easiest way to determine whether an actuator is suitable for this application is to use it. In Figure 4, we show results of measurements of a microchip surface using both systems. We can see that the systems are suitable for SPM imaging, even if the mechanical combination of the *xy* positioning systems with AFM head was very far from the optimum and we can still see some noise and thermal drifts.

The aim of this article was to present the positioning control approach, not to compare the developed stages to other systems. However, if we compare both stages to an existing commercial system for large-area SPM scanning (described in [10]), it is important to note that they offer a similar range, even if their cost is several orders of magnitude lower. On the other side, the total positioning uncertainty of commercial stages is smaller by approximately an order than for the two presented stages as the presented proof-of-concept systems were still not optimized for minimising mechanical and thermal drifts and mechanical vibrations.

## Conclusion

We show that the piezoceramic components in the *xy* stage can be efficiently and easily replaced by voice coil scanners. For the proposed control algorithm, it is, however, important to preserve the counter-force of the scanner as low as possible. This is in contrast to the typical situation in the SPM instrumentation, where all the parts are manufactured with the highest stiffnesses and resonance frequencies possible. As the presented fuzzy logic algorithm is adaptively changing the virtual system stiffness by instantly adding a force necessary for position stabilisation, the positioning stage can be operated easily far above the natural resonant frequency of the used two-dimensional guidance system.

It is also shown that the presence of a guidance system which is a must for a voice coil actuator-based system is a benefit for the metrological properties of the system. For both presented guidance systems, the systematic motion errors are below typical values observed in a system based on a stack piezoceramic actuator that is often used in the SPM instrumentation.

## Competing interests

The authors declare that they have no competing interests.

## Author’s contributions

PK carried out the development of the digital parts of the electronics and the measurement algorithm. MV developed the analog electronics and performed the atomic force microscopy measurements on the long-range SPM. VD did the design and the mechanical construction of the small *xy* scanner. JS prepared the special optical elements for the interferometers. All authors read and approved the final manuscript.

## Authors’ information

PK is the head of the Department of Nanometrology at the Czech Metrology Institute (CMI). He is working in the field of quantitative SPM, including modeling of different probe-sample interactions in micro- and nanoscale regime. MV is working at the Department of Nanometrology at CMI and is working in the field of SPM hardware and electronics development and software development. VD is the head of the Length Department at CMI. He is an expert in the field of laser interferometry, dimensional metrology and uncertainty calculation. JS is working as a research scientist at the Institute of Scientific Instruments of the Academy of Sciences of the Czech Republic. His research interests comprise magnetron sputtering, multilayer coatings, nanostructured thin films, etc.
